# Distinctive gene expression patterns in pregnancy-associated breast cancer

**DOI:** 10.3389/fgene.2022.850195

**Published:** 2022-08-10

**Authors:** Dan Wang, Huiyu Peng, Yuyao Hu, Xue Piao, Dianshuai Gao, Yan Sha

**Affiliations:** ^1^ School of Medical Information and Engineering, Xuzhou Medical University, Xuzhou, China; ^2^ The Key Laboratory of BioMedical Diagnostics, Suzhou Institute of Biomedical Engineering and Technology, Suzhou, China; ^3^ School of Life Science, Xuzhou Medical University, Xuzhou, China; ^4^ Research Center for Neurobiology of Xuzhou Medical University, Xuzhou, China

**Keywords:** breast cancer, expression profiling, immune, pregnancy, GEO

## Abstract

Pregnancy-associated breast cancer (PABC) is diagnosed during pregnancy or within 1 year postpartum, but the unique aspects of its etiology and pathogenesis have not been fully elucidated. This study aimed to ascertain the molecular mechanisms of PABC to facilitate diagnosis and therapeutic development. The Limma package was used to characterize the differentially expressed genes in PABC as compared to non-pregnancy-associated breast cancer (NPABC) and normal breast tissue. A total of 871 dysregulated genes were identified in the PABC versus NPABC groups and 917 in the PABC versus normal groups, with notable differences in the expression of MAGE and CXCL family genes. The dysregulated genes between the PABC and normal groups were mainly associated with signal transduction and immune response, while Kyoto Encyclopedia of Genes and Genomes analysis revealed that the dysregulated genes were enriched in immune-related pathways, including the major histocompatibility complex (MHC) class II protein complex, the type I interferon signaling pathway, regulation of α-β T-cell proliferation, and the T-cell apoptotic process. Through protein-protein interaction network construction, CD44 and BRCA1 were identified as prominent hub genes with differential expression in PABC versus NPABC. Furthermore, a cluster with eleven hub genes was identified in PABC versus normal adjacent tissues, of which the expression of EGFR, IGF1, PTGS2, FGF1, CAV1, and PLCB1 were verified to be differentially expressed in an independent cohort of PABC patients. Notably, IGF1, PTGS2, and FGF1 were demonstrated to be significantly related to patient prognosis. Our study reveals a distinctive gene expression pattern in PABC and suggests that IGF1, PTGS2, and FGF1 might serve as biomarkers for diagnosis and prognosis of PABC.

## Introduction

Breast cancer is the most common malignancy in women, with the frequency rising with increasing age (Smith et al., 2003; Van et al., 2010). An increase in the incidence of breast cancer over the past several decades is thought to relate to a greater number of women who delay child-bearing (Andersson et al., 2009; Raphael, Trudeau and Chan 2015). Conventionally, pregnancy-associated breast cancer (PABC) is a rare type of breast cancer that is diagnosed during pregnancy or within the following year (Lee, Mayer and Partridge 2017). The incidence of PABC is approximately 15–35 per 100,000 deliveries, with more cases diagnosed during the first postpartum year (Smith et al., 2003; Woo, Yu and Hurd 2003). The remodeling of the mammary gland to its pre-pregnant state after pregnancy might cause the mammary microenvironment to become tumor-promoting (Pepper 2006). Furthermore, diagnosis of PABC is complicated by physiological changes that accompany pregnancy. PABC generally presents as a painless palpable mass, skin changes including thickening, or bloody nipple discharge, which are often mistaken for pregnancy symptoms. Lack of detection, hesitation to proceed with medical tests, and limitations of imaging during pregnancy often result in delays in diagnosis of PABC (O’Neill et al., 2016). Therefore, it is important to improve detection and therapy of PABC.

The immune system can prevent tumor progression through immune surveillance mechanisms; however, immunogenic phenotypes in breast cancer that promote tumor growth may arise (Dunn et al., 2002). Besides, CD88^−^CD1c+CD163+ DCs (called DC3s) infiltrated luminal breast cancer primary tumors *in vivo* and DC3s was regarded to have strong potential to regulate tumor immunity (Bourdely et al., 2020). Thus, infiltrating immune cells may be both prognostic and predictive of response to breast cancer therapy (Cimino-Mathews, Foote and Emens 2015). Breast cancer can be subdivided into intrinsic molecular subtypes based on differential gene expression profiles (Perou et al., 2012; Sorlie et al., 2001; Sorlie et al., 2003; Nielsen et al., 2004). Furthermore, recent studies suggest that PABC may have a unique genomic signature associated with increased hormone levels in pregnancy (Harvell et al., 2013). Therefore, understanding the regulatory pathways and pathogenesis of breast cancer may provide a blueprint for effective immunotherapy. Nevertheless, there have been few studies that elucidate the role of the immune system in PABC, making it a challenge to design effective therapeutic strategies.

In this study, we applied microarray technology and comprehensive bioinformatics analysis to characterize gene expression profiles of PABC and compared the results with non-pregnancy-associated breast cancer (NPABC). The results characterize a distinctive gene expression pattern in PABC, which might be used in clarifying the pathogenesis of PABC and exploring a possible prognostic biomarker for early diagnosis of PABC.

## Materials and methods

### Data curation and reprocessing

Microarray data of PABC from the University of Colorado Cancer Center Tissue Bank was obtained from the Gene Expression Omnibus (GEO, http://www.ncbi.nlm.nih.gov/geo/) database under the accession number GSE31192 (Harvell et al., 2013). Transcriptome of 28 pairs of samples, including 12 tumor epithelial cells and tumor-associated stromal cells in patients with PABC (PABC group), eight tumor epithelial cells and tumor-associated stromal cells in patients with NPABC (NPABC group), and eight normal epithelial cells in patients with PABC (normal group) were used to analyze the differentially expressed genes (DEGs) using the Limma package. Dysregulated genes were screened using fold change (FC) filtering and were further selected according to the false discovery rate (FDR)-adjusted *p-value* threshold.

### GO enrichment and KEGG pathway analysis

The Gene Ontology (GO) project, which incorporates three ontologies (biological processes, cellular components and molecular functions), can be used to characterize the biological functions of sets of genes. Dysregulated genes in PABC were input into the Database for Annotation, Visualization and Integrated Discovery (DAVID; http://david.abcc.ncifcrf.gov/) to identify biological processes, cellular components, and molecular functions and *p* < 0.01 was adopted to determine the significance level. The Kyoto Encyclopedia of Genes and Genomes (KEGG, http://www.genome.ad.jp/kegg/) database was also used to analyze the potential functions of dysregulated genes and genetic pathways in PABC. Pathways with FDR < 0.05 were considered significantly enriched.

### Protein-protein interaction network construction and topology attribute analysis

The Search Tool for the Retrieval of Interacting Genes/Proteins (STRING, http://string-db.org/) database was used to retrieve predicted interactions for the identified dysregulated genes in PABC. The strength of protein interactions was set at > 0.4 as a threshold for analysis. Protein-protein interactions were analyzed by Cytoscape 3.6.1 (http://cytoscape.org/), which integrates the PPI network into attribute data and implements topology analysis.

### qRT-PCR analysis of tissue specimens from an independent cohort of PABC patients

A total of 13 pairs of PABC specimens were collected at the Affiliated Hospital of Xuzhou Medical University. Informed consent was obtained from each of the participants prior to sample collection. TRIzol reagent (Invitrogen) was utilized to extract total RNA from the tissue specimens according to the manufacturer instructions. Then, Reverse-transcribed complementary DNA was synthesized and used for the qRT-PCR. The primer sequences used for PCR amplification are shown in Supplementary Table S1.

### Statistial analysis and drawing methods

The R package Limma were used to characterize the DEGs using a threshold of |Log_2_FC| ≥ 1 (i.e., ≥2-fold difference) and the FDR-adjusted *p-value* of 0.05. The ComplexHeatmap package was used to draw heat maps. Prognostic information analyzed through the Kmplot database (http://kmplot.com/analysis). Kaplan-Meier method was used to summarize the cumulative survival rates and *p* < 0.01 was adopted to determine the significance level. Log-rank test was used to determine the association of high/low expression of hub genes with clinical characteristics. Fisher exact test was adopted to compare categorical variables.

## Results

### Gene expression profiling of a differential gene expression signature in PABC

**FIGURE 1:**
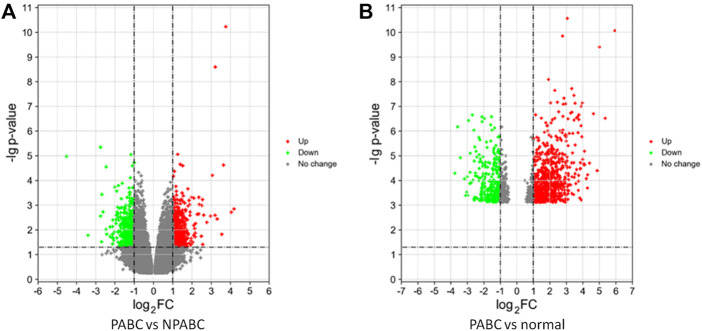
Differentially expressed genes in PABC. **(A)**. Volcano plot of the *p* values as a function of weighted fold-change for genes in PABC (*n* = 12) versus NPABC (*n* = 8) samples. Dark dots represent genes that are not significantly differentially expressed (fold change < 2, *p* < 0.05), red dots represent significantly upregulated genes (fold change ≥ 2, *p* < 0.05), and green dots represent significantly downregulated genes (fold change ≤ 1/2, *p* < 0.05). **(B)**. Volcano plot of the *p* values as a function of weighted fold change for genes in PABC (*n* = 12) versus normal (*n* = 8) samples. Dark dots represent genes that are not significantly differentially expressed (fold change < 2, *p* < 0.05), red dots represent significantly upregulated genes (fold change ≥ 2, *p* < 0.05), and green dots represent significantly downregulated genes (fold change ≤ 1/2, *p* < 0.05). Data were obtained from GEO database accession number GSE31192.

To identify genes that are differentially expressed in PABC, we performed Volcano analysis of microarray data from GEO database using a threshold of |Log_2_FC| ≥ 1 and FDR ≤ 0.05. A total of 871 DEGs were identified in PABC versus NPABC tissues, including 461 that were upregulated and 410 that were downregulated (Figure 1A). Furthermore, 917 DEGs were identified in PABC versus normal adjacent tissue, including 651 that were upregulated and 266 that were downregulated (Figure 1B). The 40 most highly differentially regulated genes (including 20 upregulated and 20 downregulated) for each of the comparisons are presented in Tables 1, 2. Notably, this includes MAGE family oncogenes (Li et al., 2021) A6/A3, A2B/A2 and A12, which were more highly expressed in PABC than in NPABC; and the CXCL family of chemokines (Palacios-Arreola et al., 2014), which were upregulated (CXCL2) or downregulated (CXCL10, CXCL11) in PABC versus normal tissues.

**TABLE 1:** List of genes with the most prominent difference in expression between the PABC and the NPABC groups.

Upregulated genes			Downregulated genes		
ID	Gene	Log_2_FC	ID	Gene	Log_2_FC
209942_x_at	**MAGEA6///MAGEA3**	4.19	205242_at	CXCL13	−4.52
214612_x_at	**MAGEA6**	4.04	209728_at	HLA-DRB4	−3.40
223687_s_at	LY6K	3.76	226147_s_at	PIGR	−2.75
213492_at	COL2A1	3.64	1559186_at	PRKXP1	−2.75
205509_at	CPB1	3.55	202018_s_at	LTF	−2.70
214603_at	**MAGEA2B///MAGEA2**	3.31	205890_s_at	UBD///GABBR1	−2.68
230910_s_at	LOC100288181	3.21	206378_at	SCGB2A2	−2.67
1553830_s_at	**MAGEA2B///MAGEA2**	3.2	228010_at	PPP2R2C	−2.62
217404_s_at	COL2A1	3.06	233030_at	PNPLA3	−2.47
210467_x_at	**MAGEA12**	2.97	1559188_x_at	PRKXP1	−2.46
205440_s_at	NPY1R	2.77	214087_s_at	MYBPC1	−2.45
239153_at	HOTAIR	2.61	210356_x_at	MS4A1	−2.43
210297_s_at	MSMB	2.56	206799_at	SCGB1D2	−2.37
203425_s_at	IGFBP5	2.55	217418_x_at	MS4A1	−2.25
218824_at	PNMAL1	2.52	1569788_at	ST8SIA1	−2.25
1562821_a_at	DSCAM-AS1	2.49	215217_at	IGKC	−2.21
207430_s_at	MSMB	2.45	216191_s_at	TRDV3	−2.15
209173_at	AGR2	2.45	206622_at	TRH	−2.13
220445_s_at	CSAG2///CSAG3	2.38	239237_at	TRG-AS1	−2.13
204942_s_at	ALDH3B2	2.36	209498_at	CEACAM1	−2.13

*Abbreviation: FC, fold change; MAGE, genes are highlighted in bold font.

**TABLE 2:** List of genes with the most prominent difference in expression between the PABC and the normal groups.

Upregulated genes			Downregulated genes		
ID	Gene	Log_2_FC	ID	Gene	Log_2_FC
223623_at	C2orf40	5.94	211122_s_at	**CXCL11**	−3.76
209560_s_at	DLK1	5.36	211080_s_at	NEK2	−3.59
1552509_a_at	CD300LG	5.01	217428_s_at	COL10A1	−3.42
204213_at	PIGR	4.87	206134_at	ADAMDEC1	−3.17
206742_at	PIR-FIGF///FIGF	4.64	204926_at	INHBA	−3.11
203980_at	FABP4	4.41	204533_at	**CXCL10**	−3.03
230101_at	**CXCL2**	4.29	229538_s_at	IQGAP3	−3.00
202037_s_at	SFRP1	4.28	210163_at	**CXCL11**	−2.97
206552_s_at	TAC1	4.22	1555758_a_at	CDKN3	−2.93
202274_at	ACTG2	4.12	222608_s_at	ANLN	−2.85
228766_at	CD36	4.10	200832_s_at	SCD	−2.83
226147_s_at	PIGR	4.06	218542_at	CEP55	−2.82
209774_x_at	**CXCL2**	4.05	223278_at	GJB2	−2.77
209292_at	ID4	3.99	218404_at	SNX10	−2.69
242626_at	SAMD5	3.98	203936_s_at	MMP9	−2.65
228653_at	SAMD5	3.95	209773_s_at	RRM2	−2.60
202036_s_at	SFRP1	3.91	236313_at	CDKN2B	−2.58
202350_s_at	LOC100506558///MATN2	3.91	239002_at	ASPM	−2.58
228399_at	OSR1	3.90	237753_at	IL21R	−2.56
202965_s_at	CAPN6	3.86	204962_s_at	SLC35F6///CENPA	−2.55

*Abbreviation: FC, fold change; CXCL, genes are highlighted in bold font.

**FIGURE 2:**
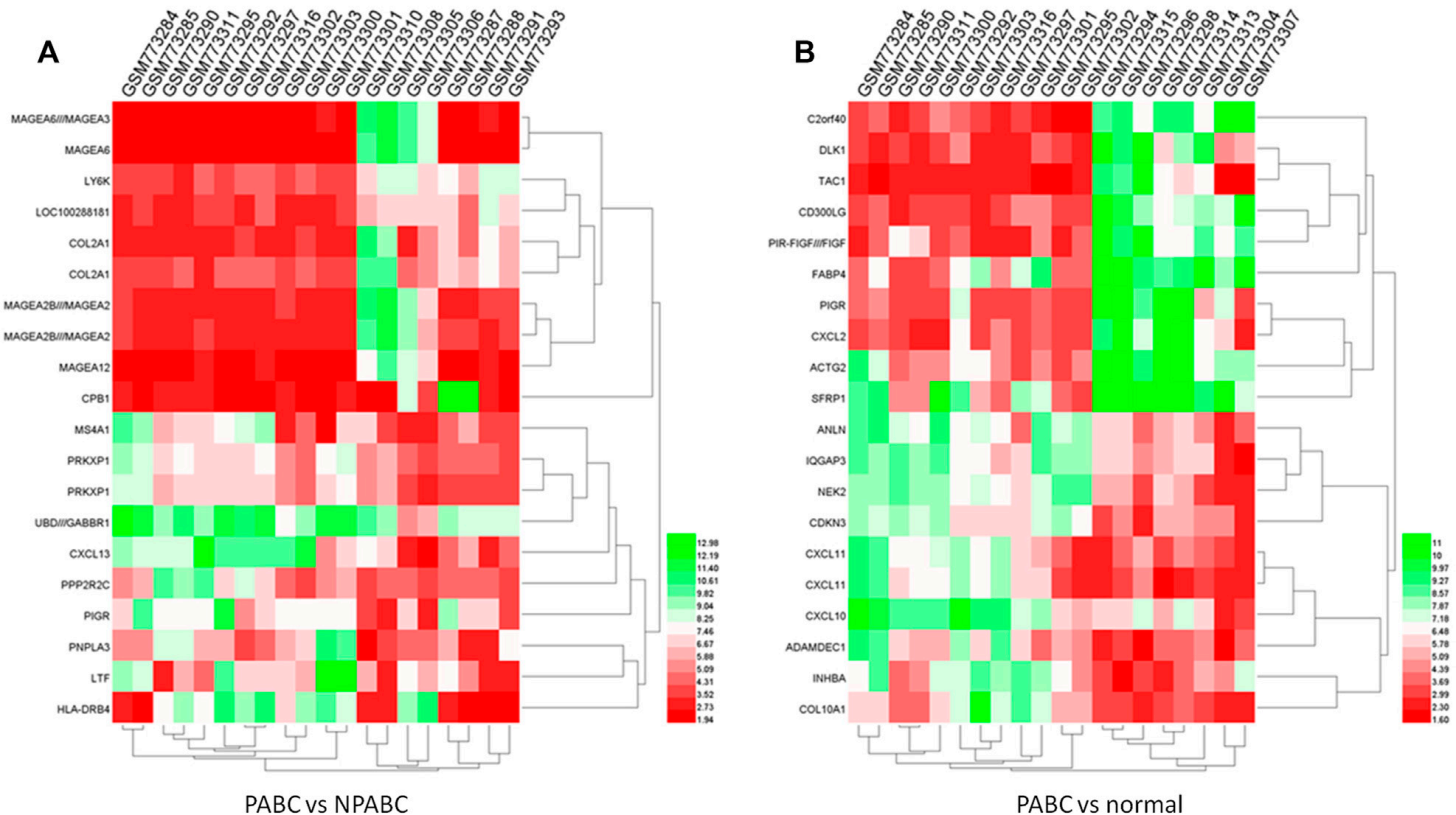
Heatmap of expression profiles for the 20 most highly dysregulated (10 upregulated and 10 downregulated genes) in PABC. The red through green color indicates high to low expression levels. The PABC samples are indicated by blue font. **(A)**. Heatmap for the top 20 DEGs in the PABC versus NPABC samples. The left 12 columns are PABC samples and the right eight columns are NPABC samples. **(B)**. Heatmap for the top 20 dysregulated genes in PABC versus normal tissues. The left 12 columns are PABC samples and the right eight columns are normal samples.

To visualize the differences in expression between PABC and either NPABC or normal tissues in greater detail, we performed heat mapping. The patterns of expression for the 20 top dysregulated genes for each comparison, including 10 upregulated and 10 downregulated genes, are shown in Figures 2A,B. The clustering patterns for the PABC samples (first 12 columns) as compared to either the NPABC samples (Figure 2A, last eight columns) or the normal group (Figure 2B, last eight columns) were clearly partitioned, with relatively discreet expression patterns between the groups. However, the samples within the NPABC group (Figure 2A, last eight columns) had a more variable pattern that reflected at least two different subtypes. A similar result was observed for heatmap that included all genes with a more stringent threshold of |Log_2_FC| ≥ 1 and FDR ≤ 0.01 (Supplementary Figure S1). These results support previous findings (Harvell et al., 2013) suggesting that PABC may represent a discreet type of breast cancer with a gene expression signature that is distinct from that of NPABC.

### GO enrichment and KEGG pathway analyses to identify unique processes that underlie PABC

To further explore the functions of DEGs in PABC, we performed GO enrichment analysis and KEGG pathway analysis. Through GO analysis, we identified 157 pathways that were significantly enriched. The dysregulated genes were associated with “signal transduction” and “the immune response” (ontology: biological process), “integral component of membrane”, “membrane and integral component of plasma membrane” (ontology: cellular component), and “protein binding” (ontology: molecular function) in the PABC group versus the NPABC group. However, the genes were most highly associated with “positive regulation of transcription from the RNA polymerase II promoter” (ontology: biological process), “nucleus” and “cytoplasm” (ontology: cellular component) and “ATP binding” (ontology: molecular function) in the PABC group versus the normal group.

Through KEGG pathway analysis, the dysregulated genes in the PABC group versus the NPABC group were mainly involved in the following pathways: 1) Tuberculosis; 2) cytokine-cytokine receptor interaction; 3) Herpes simplex infection; 4) cell adhesion molecules (CAMs); 5) Influenza A; 6) Rheumatoid arthritis and 7) the phagosome signaling pathway. The genes in the PABC group versus the normal group were mainly enriched in the following pathways: 1) pathways in cancer; 2) the PI3K-Akt signaling pathway; 3) focal adhesion; 4) the Rap1 signaling pathway; 5) the Ras signaling pathway; 6) proteoglycans in cancer; 7) regulation of actin cytoskeleton; and 8) microRNAs in cancer. Among these, pathways in cancer (hsa05200), the PI3K-Akt signaling pathway (hsa04151) and focal adhesion (hsa04510) were the top three significantly enriched networks with FDR < 0.05. Collectively, these results support unique pathways and processes that may underlie the development of PABC.

### Construction of the protein-protein interaction network for PABC

**FIGURE 3:**
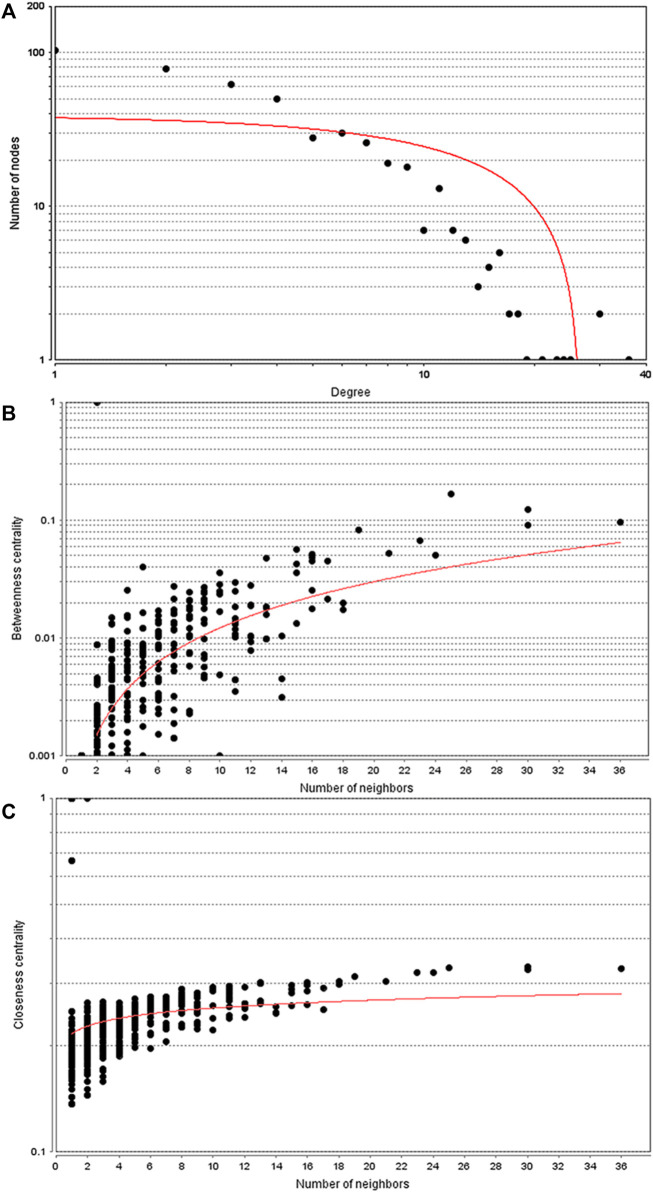
Topology parameters for the PABC differential expression PPI network. PPI network analysis was performed using 580 proteins that were differentially regulated in PABC versus NPABC as identified by STRING. **(A)** Distribution of the Nodes versus Degree. **(B)** Betweenness Centrality versus Number of Neighbors for the Nodes. **(C)** Closeness Centrality versus Number of Neighbors for the Nodes. Best fit curves are drawn in red.

Given the above evidence for a unique transcriptomic signature for PABC, we sought to use current bioinformatics knowledge to further characterize the roles for genes and their protein products. Among the 871 dysregulated proteins in the PABC group versus the NPABC group, 580 were identified in the STRING database and entered into Cytoscape 3.7.1 to construct a PPI network. The topological parameters were calculated using the R package. The main connected component included 460 nodes and 1,092 edges (Figure 3A). The top 10% of the nodes ranked by degree value (containing 46 proteins) were selected as hub nodes, and the distribution of degree, betweenness and closeness was determined (Figures 3B,C). These hub nodes and their characteristic properties are tabulated (Supplementary Table S2). Notable findings were that the cancer progression-associated genes CD44 (Ghotra et al., 2015), as well as BRCA1 (Semmler et al., 2019), a classic breast cancer gene, were highly represented in the PPI network for PABC versus NPABC. Furthermore, PTPRC/CD45 (Al Barashdi et al., 2021) and IL1B (Gelfo et al., 2020), which are associated with immune and inflammatory activity, were represented among the proteins with the highest degree value.

**FIGURE 4:**
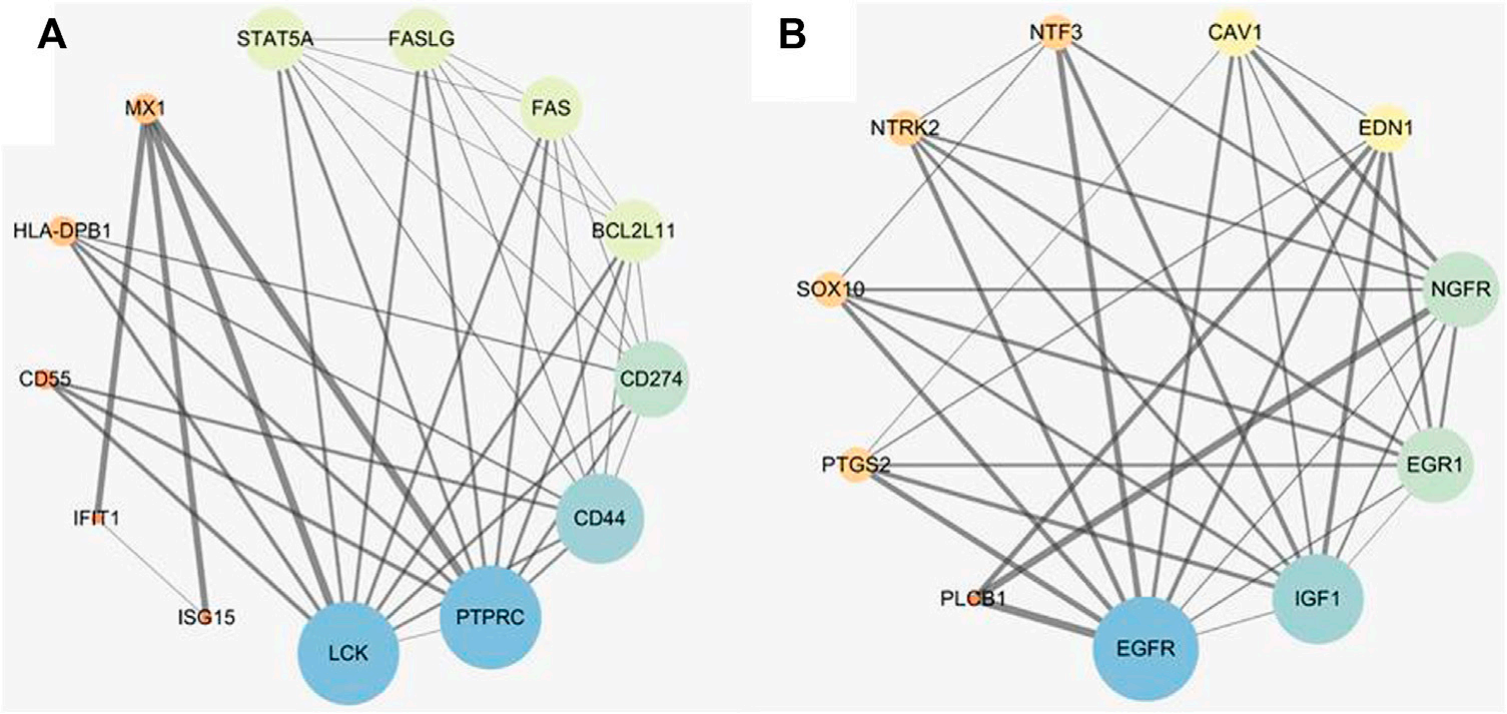
Candidate biomarker panel related to PABC including proteins and their interaction. The size of the nodes represents the degree value, with bigger sizes corresponding to higher degree values. **(A)**. Candidate biomarker panel between PABC and NPABC groups. **(B)**. Candidate biomarker panel between PABC and normal groups.

To further identify patterns within the PPI network for PABC versus NPABC, we performed additional clustering analysis. Six clusters had at least 10 nodes, among which Cluster 2 (Figure 4A) contained the largest number of proteins. Twenty-three proteins within this cluster were associated with the MHC class II protein complex (36.36%), type I interferon signaling pathway (36.36%), regulation of α-β T cell proliferation (18.18%), and the T cell apoptotic process (9.09%). Furthermore, 13 common key proteins between hub nodes and Cluster two included LCK, PTPRC, CD44, HLA-DPB1, CD274, MX1, BCL2L11, STAT5A, FAS, FASLG, CD55, IFIT1, and ISG15.

We also performed a similar analysis of the PABC versus normal groups and identified 921 dysregulated proteins. A total of 558 proteins were identified in the STRING database and used to construct a PPI network for PABC versus normal samples. The main network consisted of 290 nodes and 858 edges, and the top 10% of the node degree (containing 29 proteins) were chosen as hub nodes (Supplementary Table S3). This includes many classic oncogenic proteins, with EGFR (Sigismund et al., 2018), Myc (Dhanasekaran et al., 2022) and Jun (Vleugel et al., 2006) having the highest node degrees. Only one statistically validated cluster was identified by the presence of at least 10 nodes. The cluster was made up of 17 proteins, which were associated with regulation of vasoconstriction (39.47%), phospholipase C activity (21.05%), positive regulation of nuclear division (18.42%), positive regulation of smooth muscle cell proliferation (15.79%), and positive regulation of acute inflammatory response (5.26%) (Figure 4B). Eleven common key proteins including EGFR, IGF1, NGFR, EDN1, PTGS2/COX-2, EGR1, NTRK2, NTF3, CAV1, SOX10, and PLCB1 were identified between hub nodes within the cluster.

### qRT-PCR validation and survival analysis

To further validate the identification of key proteins that are dysregulated in PABC, we collected samples from an independent cohort of PABC patients, including paired PABC and normal breast tissues. Next, we performed qRT-PCR to evaluate the mRNA expression of the 11 hub proteins identified by PPI network analysis of PABC versus normal tissues (Figure 4B). The results demonstrate that the expression of EGFR, IGF1, PTGS2, FGF1, CAV1, and PLCB1 were significantly different (*p* < 0.01) in PABC tissues as compared to non-tumor tissues, with a similar pattern to that observed in the microarray analysis. GO and KEGG pathway analyses showed that these six genes are involved in pathways in multiple cancers, the PI3K-Akt signaling pathway, focal adhesion, Rap1 signaling pathway, and Ras signaling pathway, suggesting that they may play an important role in the carcinogenesis of PABC. The prognostic information of the six hub genes was analyzed through the Kmplot database (http://kmplot.com/analysis). Statistical difference (*p* < 0.01) in OS (Overall Survival) was observed in three of the six hub genes, including IGF1, PTGS2, and FGF1, thus further highlighting their potential relevance in PABC.

## Discussion

Numerous studies have attempted to characterize the expression profile in breast cancer to identify biomarkers for diagnosis, prognosis or personalized therapy (Liu et al., 2017; Zhang et al., 2018). Furthermore, PABC has been recognizes as a distinct form of breast cancer with unique properties (Pepper, 2006; Andersson et al., 2009; Lee et al., 2017). A 2013 study evaluated PABC transcriptomic signatures from the GSE31192 dataset (Harvell et al., 2013), and a more recent study integrated two microarray profile datasets (GSE31192 and GSE53031) to identify core genes and clinical roles in PABC through integrated bioinformatic analysis (Zhang et al., 2019). The GSE31192 and GSE53031 datasets were from different sample sources, including cancer epithelial tissues, cancer stroma tissues, normal epithelial tissues, and normal stroma tissues. Therefore, data were grouped according to the sample sources. However, in this study, we compared PABC with both NPABC samples and normal samples regardless of the tissue source, which may give us a more holistic assessment of signature features that are specific to PABC regardless of the epithelial versus stromal sample source.

We identified 871 DEGs for the PABC versus NPABC group comparison and 917 DEGs for the PABC versus normal group comparison. Among the most highly dysregulated genes, the MAGE family oncogenes, including MAGE A6/A3, A2B/A2 and A12, were found to be expressed at elevated levels in PABC. Given the association of MAGE proteins with extremely aggressive tumor types (Li et al., 2021), these findings may underscore the aggressive PABC phenotype. We also determined that CXCL2 is upregulated and CXCL10 and 11 are downregulated in PABC versus adjacent normal tissues. These proteins belong to a family of chemokines that are associated with proliferation and angiogenesis in breast cancer and have been explored as potential therapeutic targets (Palacios-Arreola et al., 2014).

To further understand the patterns of differential expression, we generated heatmaps of the most highly dysregulated genes, which demonstrated that samples of the PABC group were clearly separated from those of the NPABC and normal groups. Gene ontology and enrichment analyses further indicated that the DEGs in PABC versus NPABC were mainly enriched in pathways in cancer, the PI3K-Akt signaling pathway, focal adhesion, the Rap1 signaling pathway, proteoglycans in cancer, microRNAs in cancer, prostate cancer, the p53 signaling pathway, small cell lung cancer, and melanoma and glioma pathways. Many studies have demonstrated dysregulation in the PI3K-AKT pathway in cervical cancer, breast cancer, malignant glioma, and other cancers (Lee et al., 2015; Guerrero-Zotano, 2016; Mayer and Arteaga; Li et al., 2016), thus suggesting that the PI3K-AKT pathway may be an important contributor to the oncogenic PABC phenotype.

For more in-depth analysis of functional pathways associated with DEGs in PABC, we also performed PPI network analysis and clustering (Berkhin 2006). Interestingly, the classic breast cancer marker genes, BRCA1 (Semmler et al., 2019) and CD44 (Chen et al., 2018) were overexpressed in the PABC PPI network, suggesting that PABC may be characterized by higher activity of proteins that contribute to NPABC. Our results also emphasize the role for immune and inflammatory pathways in PABC. Notably, the well-established immune pathway activators PTPRC/CD45 and IL1B (Sigismund et al., 2018; Al Barashdi et al., 2021) were among the proteins with the highest node degree value in the corresponding PPI network. According to the clustering analysis, six clusters of proteins were dysregulated, and the largest cluster was associated with the MHC class II protein complex (36.36%), type I interferon signaling pathway (36.36%), regulation of α-β T-cell proliferation (18.18%) and T-cell apoptotic process (9.09%). MHC class II is known to be downregulated in many metastatic tumors, including human breast cancer cells (Shi et al., 2006). On the other hand, expression of MHC-II in melanoma is associated with an intensified response to PD-1-targeted immunotherapy (Johnson et al., 2016), which has been subsequently validated in classic Hodgkin lymphoma (Roemer et al., 2018) and with combination immunotherapy (Rodig et al., 2018). MHC-II expression can characterize T-cell-inflamed or immune-responsive subset of tumors (Balko et al. 2018). The association of type I interferon within the PPI may be indicative of a complex relationship between stress and immune function that has been reported in breast cancer (Mundy-Bosse et al., 2011). Type I interferon is the prototype member of a class of antiviral immunomodulatory cytokines involved in tumor initiation and progression and can act on tumor cells directly by inhibiting cell growth or indirectly by activating immune cells to mount antitumor responses (Zitvogel et al., 2015). Metastatic progression is the major cause of breast cancer-related mortality, and in multiple models of breast cancer, dysregulated immunity due to the loss of host type-I IFN signaling may drive metastasis (Rautela et al., 2015). A role for T-cells in the PABC phenotype, as indicated by our clustering analysis, is also consistent with mechanisms that are known to impact breast cancer progression. Higher levels of T cells at early stages of differentiation have been detected in breast cancer patients (*p* < 0.05) (Speigl et al., 2017), and CD14^+^ myeloid cells from breast cancer patients have been shown to have enhanced ability to suppress autologous T-cell proliferation (Speigl et al., 2017). Furthermore, interleukin 10 and interleukin two synergistically function to promote cytotoxicity of CD8^+^ T-cell, which is inhibited by regulatory T cells in breast cancer (Li et al., 2017). Thus, increased understanding of functional pathways and molecular mechanisms will provide a blueprint for effective immunotherapy for breast cancer, and for PABC in particular.

Our PPI network analysis of PABC versus normal breast tissue samples also identified a single cluster for which EGFR, IGF1, PTGS2, FGF1, CAV1 and PLCB1 were verified to be differentially expressed in an additional independent cohort of patients’ PABC tissue compared with normal tissue. EGFR has been demonstrated to regulate epithelial tissue development and homeostasis in a variety of types of cancer, including breast cancer (Sigismund et al., 2018), with approximately half of triple-negative breast cancer and inflammatory breast cancer cases overexpressing EGFR (Masuda et al., 2012). Furthermore, the IGF1 signaling pathway has previously been shown to be critical for normal cell division and to be modulated in PABC (Loddo et al., 2014). Targeted therapies have been developed for suppressing the post-partum pro-tumorigenic extracellular matrix *via* inhibition of PTGS2 (O'Brien et al., 2011), and the potential benefit of targeting the FGF1 pathway in breast cancer has been considered (Francavilla and O’Brien, 2022). On the other hand, CAV1 expression, which is down-regulated during tumorigenesis, has been shown to be suppressed during lactation (Park et al., 2001). Therefore, the identification of these proteins within a PPI cluster is consistent with known mechanisms in breast cancer that may be modulated during the pregnancy and postpartum periods. Notably, three of these genes, including IGF1, PTGS2, and FGF1, were significantly related to breast cancer patient outcomes, thus further verifying their potential roles as prognostic biomarkers.

## Conclusion

In this study, we screened differentially expressed genes from microarray datasets of PABC by using an integrated bioinformatics analysis approach. A distinctive pattern of cancer and immune-related gene expression revealed a landscape of PABC-associated genes and an interaction network of their protein products, suggesting that these genes may have a distinct biological nature in PABC. Furthermore, EGFR, IGF1, PTGS2, FGF1, CAV1, and PLCB1, were verified to be expressed differentially in PABC compared with normal tissues, and IGF1, PTGS2, and FGF1 were demonstrated to be significantly related to breast cancer patient prognosis, suggesting that the three genes might serve as biomarkers for precision diagnosis and treatment of PABC.

## Data Availability

The original contributions presented in the study are included in the article/Supplementary Material, further inquiries can be directed to the corresponding author.
