# Supplementary material for: Distinctive gene expression patterns in pregnancy-associated breast cancer
Source: Front Genet. 2022 Aug 10;13:850195. doi: 10.3389/fgene.2022.850195 (PMC9399642; doi:10.3389/fgene.2022.850195)
Supplement: Supplementary file 1 [file DataSheet1.doc]

Table S1. Primers used in qRT-PCR for detecting gene expression.

| Gene name | Primer sequence(5'-3') |
| --- | --- |
| EGFR | F:GGAGGTGGCTGGTTATGTCC |
|  | R:TGTGATAATTCAGCTCAAACCTG |
| IGF1 | F:AGCTCTCTAGGGAAAGAGGTGA |
|  | R:AGCTCTCTAGGGAAAGAGGTGA |
| NGFR | F:CCGTTGGATTACACGGTCCA |
|  | R:AGCATCGGTTGTCGGAATGT |
| EDN1 | F:CCCGTTAAAAGGGCACTTGGG |
|  | R:GTTTTGAACGAGGACGCTGG |
| PTGS2 | F:GGCCATGGGGTGGACTTAAA |
|  | R:GCTCTGGTCAATGGAAGCCT |
| EGR1 | F:CCCCGACTACCTGTTTCCAC |
|  | R:CTGTCATGTCCGAAAGCCCT |
| NTRK2 | F:GGAATTGGGTTGGAGCAGGA |
|  | R:TTGCCCACCAGGATCAGTTC |
| NTF3 | F:GCTCCTCTCCCTTCCGAAC |
|  | R:CTATCCGTATCCACCGCCAG |
| CAV1 | F:ACGTAGACTCGGAGGTAGGC |
|  | R:CGGATGGGAACGGTGTAGAG |
| SOX10 | F:CCAGGGTGGTTGGTGGTAAG |
|  | R:AAGTGGGCGCTCTTGTAGT |
| PLCB1 | F:TGGCTGCTTTGACACTGGAA |
|  | R:CTGCAGCTTGGGCTTTTCAT |

Table S2. The top 10% of the nodes (including 46 proteins) based on the degree value for the PABC group versus the NPABC group were selected as hub nodes. D, BC, CC, S, and ASPL correspond to the degree, betweenness centrality, closeness centrality, stress and average shortest path lengths, respectively.

| Protein name | D | BC | CC | S | ASPL |
| --- | --- | --- | --- | --- | --- |
| PTPRC | 36 | 0.10 | 0.33 | 102642 | 3.03 |
| CD44 | 30 | 0.09 | 0.33 | 92272 | 3.00 |
| IL1B | 30 | 0.12 | 0.33 | 107674 | 3.05 |
| BRCA1 | 25 | 0.17 | 0.33 | 126484 | 3.02 |
| LCK | 24 | 0.05 | 0.32 | 56076 | 3.11 |
| STAT5A | 23 | 0.07 | 0.32 | 70572 | 3.11 |
| ISG15 | 21 | 0.05 | 0.30 | 47272 | 3.30 |
| IRS1 | 19 | 0.08 | 0.31 | 71046 | 3.20 |
| CD274 | 18 | 0.02 | 0.30 | 25058 | 3.30 |
| MX1 | 18 | 0.02 | 0.30 | 19088 | 3.37 |
| HLA-DPB1 | 17 | 0.02 | 0.29 | 26604 | 3.44 |
| FBXO7 | 17 | 0.05 | 0.25 | 34632 | 3.96 |
| CD55 | 16 | 0.05 | 0.30 | 38964 | 3.38 |
| BCL2L11 | 16 | 0.03 | 0.30 | 29638 | 3.32 |
| ANAPC10 | 16 | 0.05 | 0.28 | 33870 | 3.51 |
| TRIM28 | 16 | 0.05 | 0.26 | 32638 | 3.84 |
| FASLG | 16 | 0.02 | 0.30 | 28260 | 3.36 |
| POLR2I | 15 | 0.04 | 0.28 | 31218 | 3.55 |
| FBXL7 | 15 | 0.01 | 0.26 | 13294 | 3.87 |
| NCK1 | 15 | 0.04 | 0.30 | 35752 | 3.38 |
| GRIA1 | 15 | 0.06 | 0.29 | 37824 | 3.48 |
| KLHL22 | 14 | 0.00 | 0.25 | 6144 | 4.05 |
| KLHL13 | 14 | 0.00 | 0.25 | 5014 | 4.02 |
| DCTN5 | 14 | 0.01 | 0.26 | 10740 | 3.89 |
| TLR10 | 13 | 0.01 | 0.30 | 15428 | 3.34 |
| TLR6 | 13 | 0.01 | 0.30 | 15428 | 3.34 |
| AREL1 | 13 | 0.02 | 0.26 | 14244 | 3.81 |
| DYNC1I1 | 13 | 0.02 | 0.27 | 12066 | 3.73 |
| AURKA | 13 | 0.05 | 0.30 | 40858 | 3.34 |
| CASP1 | 13 | 0.02 | 0.30 | 19278 | 3.32 |
| TRIM36 | 12 | 0.01 | 0.24 | 9774 | 4.17 |
| CAPZA1 | 12 | 0.01 | 0.26 | 7398 | 3.89 |
| HNRNPU | 12 | 0.03 | 0.25 | 23868 | 3.93 |
| IFIT1 | 12 | 0.01 | 0.26 | 12626 | 3.80 |
| FAS | 12 | 0.01 | 0.29 | 17568 | 3.42 |
| COPS6 | 12 | 0.02 | 0.26 | 24028 | 3.82 |
| GPS1 | 12 | 0.02 | 0.26 | 23802 | 3.81 |
| IL6R | 11 | 0.01 | 0.29 | 14164 | 3.41 |
| CSF3R | 11 | 0.01 | 0.28 | 14566 | 3.53 |
| IGHV4-38-2 | 11 | 0.01 | 0.27 | 10766 | 3.69 |
| RNF19A | 11 | 0.00 | 0.24 | 3840 | 4.19 |
| IL23A | 11 | 0.01 | 0.29 | 16764 | 3.47 |
| GMPS | 11 | 0.03 | 0.28 | 24286 | 3.58 |
| UBE2D4 | 11 | 0.00 | 0.24 | 2520 | 4.12 |
| RNF41 | 11 | 0.01 | 0.26 | 11970 | 3.89 |
| NDE1 | 11 | 0.02 | 0.27 | 16572 | 3.65 |

Table S3. The top 10% of the nodes (including 29 proteins) based on the degree value for the PABC group versus the normal group were selected as hub nodes. D, BC, CC, S, and ASPL correspond to degree, betweenness centrality, closeness centrality, stress, and average shortest paths length, respectively.

| Protein name | D | BC | CC | S | ASPL |
| --- | --- | --- | --- | --- | --- |
| EGFR | 51 | 0.26 | 0.43 | 81388 | 2.32 |
| MYC | 42 | 0.14 | 0.40 | 50238 | 2.48 |
| JUN | 38 | 0.12 | 0.40 | 43904 | 2.50 |
| IGF1 | 36 | 0.10 | 0.41 | 40432 | 2.44 |
| KIT | 25 | 0.03 | 0.37 | 14706 | 2.73 |
| NTRK2 | 23 | 0.05 | 0.37 | 19652 | 2.68 |
| EGR1 | 23 | 0.03 | 0.37 | 12604 | 2.68 |
| EDN1 | 22 | 0.04 | 0.37 | 18036 | 2.68 |
| MYLK | 21 | 0.02 | 0.32 | 8180 | 3.08 |
| NTF3 | 19 | 0.02 | 0.35 | 8406 | 2.85 |
| NGFR | 19 | 0.01 | 0.36 | 6552 | 2.81 |
| EPHA2 | 19 | 0.02 | 0.36 | 8444 | 2.80 |
| MYH11 | 19 | 0.04 | 0.33 | 12278 | 3.03 |
| PTGS2 | 19 | 0.02 | 0.37 | 9214 | 2.73 |
| FGFR1 | 18 | 0.02 | 0.36 | 7582 | 2.79 |
| ACTA2 | 18 | 0.02 | 0.33 | 6966 | 3.01 |
| CAV1 | 18 | 0.03 | 0.37 | 11220 | 2.71 |
| ACTG2 | 17 | 0.01 | 0.30 | 4478 | 3.36 |
| RELN | 17 | 0.04 | 0.34 | 14720 | 2.96 |
| NES | 17 | 0.02 | 0.36 | 9618 | 2.75 |
| EFNB2 | 16 | 0.05 | 0.35 | 18976 | 2.83 |
| MYL9 | 16 | 0.00 | 0.30 | 2566 | 3.31 |
| TPM2 | 16 | 0.02 | 0.31 | 4738 | 3.19 |
| EFNB3 | 14 | 0.01 | 0.32 | 6856 | 3.11 |
| TCF7L2 | 14 | 0.02 | 0.32 | 9572 | 3.12 |
| LRP5 | 14 | 0.02 | 0.33 | 7704 | 3.00 |
| PAK3 | 14 | 0.05 | 0.34 | 16310 | 2.93 |
| PLCB1 | 14 | 0.02 | 0.34 | 10396 | 2.96 |
| SOX10 | 14 | 0.02 | 0.36 | 7488 | 2.78 |

**Figure Legends:**

Figure S1. Heatmap for the genes in the PABC versus NPABC samples with a threshold of |Log_2_FC| ≥ 1 and FDR ≤ 0.01. The left 12 columns are PABC samples and the right 8 columns are NPABC samples.
