# Supplementary figures and images for: Distinctive gene expression patterns in pregnancy-associated breast cancer
Source: Front Genet. 2022 Aug 10;13:850195. doi: 10.3389/fgene.2022.850195 (PMC9399642; doi:10.3389/fgene.2022.850195)

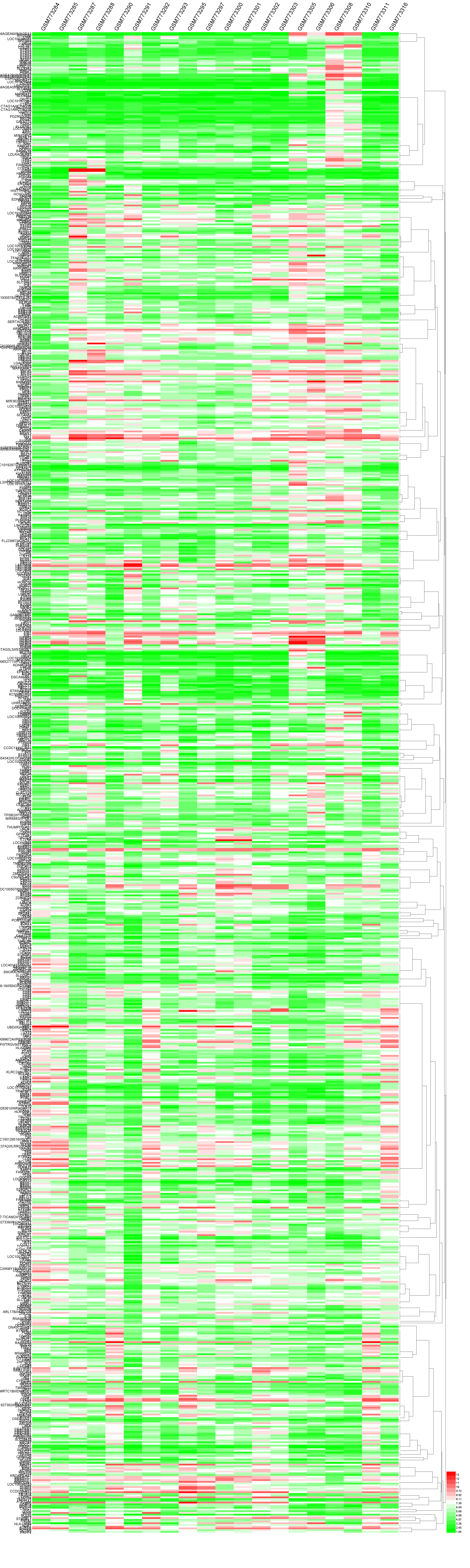

Supplement: Supplementary file 2 [file Image1.PNG]
